# Laboratory Markers in the Prediction of Acute Perforated Appendicitis in Children

**DOI:** 10.1155/2019/4608053

**Published:** 2019-09-19

**Authors:** Jing Yang, Chong Liu, Yuxia He, Zhangqiao Cai

**Affiliations:** ^1^Emergency Department, Affiliated Renhe Hospital of China Three Gorges University, Yichang, Hubei, China; ^2^Department of Neurology, Yiling Hospital, Yichang, Hubei, China; ^3^Department of Otolaryngology, Affiliated Renhe Hospital of China Three Gorges University, Yichang, Hubei, China

## Abstract

**Objective:**

This study aimed to explore the laboratory markers associated with perforation in children with acute appendicitis.

**Methods:**

This retrospective study reviewed 1895 children (3–18 years old) with confirmed acute appendicitis from 2007 to 2017. Clinical (demographic characteristics, symptoms, and signs) and laboratory data (white blood cell count, C-reactive protein (CRP), procalcitonin, D-lactate, platelet count, bilirubin, aspartate aminotransferase, and alanine aminotransferase) were collected and compared between perforated and nonperforated groups. The logistic regression analysis was performed to identify independent risk factors.

**Results:**

Of all patients, 613 children were perforated. Children with perforation had significantly longer duration of symptoms, higher white blood cell count, CRP level, and neutrophils percentage, and lower serum sodium level. Elevated white blood cell count with CRP level and elevated neutrophils percentage with CRP level were found to be associated with risk of perforation.

**Conclusions:**

White blood cell count with C-reactive protein and neutrophils percentage with CRP are important markers in distinguishing perforated appendicitis from nonperforated appendicitis in pediatric subjects.

## 1. Introduction

Acute appendicitis (AA) is the most common surgery in children emergency [1]. In AA, about 30–75% of children progress to perforation, especially in children younger than 5 years [2]. Previous studies have shown that children have higher rates of perforation compared with adults [3–6]. Delayed diagnosis and treatments often increase the risk of complications, such as perforation, abscess formation, peritonitis, and partial bowel obstruction.

The main reasons for difficult diagnosis of AA in children included atypical clinical symptoms in children, varied presentation, and a wide range of differential diagnoses [7]. Despite the multiple new diagnostic methods were available, the initial misdiagnosis rates range from 28% to 57% in children younger than 12 years to nearly 100% in children younger than 2 years [8, 9]. Therefore, identifying children at risk for perforated appendicitis is important since it decides further workup and management.

Laboratory markers were less affected by subjective factors, so they may be used as a more reliable index to predict perforation. For example, the risk of perforation in pediatric appendicitis was reported to correlate with increased white blood cell count (WBC) and C-reactive protein (CRP) [10]. However, poor correlation between WBC and appendiceal perforation was found in some studies [11, 12].

The present study was undertaken to determine the laboratory markers in prediction of appendiceal perforation in AA in children.

## 2. Materials and Methods

### 2.1. Patients and Study Design

This study is a retrospective analysis of pediatric patients (younger than 18 years) diagnosed with appendicitis and treated with appendectomy from June, 2007, to June, 2017, in Emergency Department, Affiliated Renhe Hospital of China Three Gorges University, which is a tertiary care hospital. Patients with incomplete records, pregnancy, bleeding disorders, and severe anemia, chronic respiratory, cardiovascular, hepatic, or renal disease were excluded from this study. Children younger than 3 years were excluded as well because they are not able to express and localize pain reliably.

Detailed information was obtained, such as age, gender, weight, height, duration of symptoms, physical examinations, laboratory examinations (e.g., WBC, CRP, platelet count, bilirubin, serum sodium procalcitonin (PCT), D-lactate (DLAC), aspartate aminotransferase (AST), alanine aminotransferase (ALT)), and time to surgery. The laboratory test was taken on admission. This study was approved by the institutional review board of local hospital.

### 2.2. Primary Outcome

The primary outcome was the presence of perforated appendicitis, which was confirmed during surgery according to the spread of pus in the abdominal cavity, visual hole in the appendix, or the presence of an appendicolith in the abdominal cavity. The removed appendix was also sent for histopathological examination in all children. We excluded children with normal appendix.

### 2.3. Independent Variables and Their Definition

Duration of symptoms was defined as the interval from the symptoms of present illness to the final diagnosis in hospital and classified into <24 h and >24 h groups. Time to surgery was defined as the time from first evaluation in hospital to time of incision.

### 2.4. Statistical Analysis

Comparisons between perforated vs nonperforated appendicitis were performed using Student's *t*-test and Pearson's chi-square. Clinically relevant variables and variables found to be statistically significant were included in the logistic regression. The logistic regression was performed to determine the risk factors. Adjusted ORs and their 95% CI were obtained. *P* < 0.05 was considered significant.

## 3. Results

### 3.1. Demographic Characteristics

A total of 1895 children aged 3 to 18 years were finally studied after exclusion of children with normal appendix and younger than 3 years. The demographic characteristics are summarized in Table 1. There was no difference in male-female ratio, age, temperature, and BMI between the perforated and nonperforated groups.

### 3.2. Symptoms and Signs between the Perforated and Nonperforated Groups

There was no difference in various symptoms and signs and time from admission to surgery between the perforated and nonperforated groups. The duration of symptoms was longer in children with perforated appendicitis compared with nonperforated groups (Table 2).

### 3.3. Laboratory Values between the Perforated and Nonperforated Groups

The perforated group had a significantly higher value in WBC count (14,890 ± 723 vs 12,650 ± 558), CRP (8.37 ± 0.84 vs 5.79 ± 0.65), and neutrophils percentage (77.3 vs 65.1) compared with nonperforated groups. The perforated group had a significantly lower level of serum sodium (133 ± 2 vs 137 ± 2) compared with nonperforated groups. ANC, PCT, and DLAC were compared with nonperforated groups. There was no significant difference in platelet count, PCT, DLAC, bilirubin, and AST and ALT levels between the 2 groups (Table 3).

### 3.4. Risk Factors Associated with Perforated Diagnosis of Acute Appendicitis

In logistic regression analysis, our results showed that elevated white blood cell count (>12000 mm^3^) with CRP level (>8 mg/dL) and elevated neutrophils percentage (>74%) with CRP (>8 mg/dL) increased the odds of a perforated diagnosis of acute appendicitis. However, duration of symptoms, WBC count, CRP, neutrophils percentage, and serum sodium level did not increase the odds of a perforated diagnosis of acute appendicitis despite their values were significantly different between groups (Table 4).

## 4. Discussion

Appendiceal perforation, subsequent abscess formation, and panperitonitis are still common in children with appendicitis, so there is a need to establish the diagnostic value of objective markers such as laboratory data in this population. In this study, we explored the value of several common laboratory markers in predicting perforated appendicitis in children.

WBC count had been used to differentiate patients with and without appendicitis and discriminate simple from perforated appendicitis according to the time from the onset of symptoms to diagnosis. Previous study showed that WBC count and its sensitivity increased from the onset of symptoms to diagnosis [13]. CRP levels were correlated with the severity of appendiceal inflammation, and higher CRP levels were often found in more advanced disease [14]. Besides, studies also found that CRP may be sensitive in the prediction of appendiceal perforation [15]. However, several studies suggested that WBC counts and CRP were insensitive and unspecific to distinguish perforated and nonperforated AA [16, 17]. In the present study, we found that both WBC counts and CRP level were significantly higher in children with perforated AA compared with nonperforated AA. However, only combined WBC counts and CRP level can be used to predict the risk of perforation in the regression model.

Increased neutrophil percentage is often associated with bacterial infection. Previous data had confirmed the use of neutrophil percentage in predicting an appendicular perforation as a result of acute appendicitis [18]. Consistently, our data suggested that neutrophil percentage was significantly higher in children with perforated AA. However, only combined neutrophil percentage and CRP level can be used to predict the risk of perforation in the regression model. Our study suggested that elevated levels of neutrophil percentage (>74%) and CRP (>8 mg/dL) predicted that the risk of perforated appendicitis is increased more than 5 times. These patients should be strongly considered for an urgent appendectomy; otherwise, the patients may be amenable to nonoperative management with antibiotics alone.

Hyponatremia is a new predictor of perforated appendicitis. Previous study suggested that hyponatremia was more useful than WBC count in diagnosing complicated appendicitis [19]. Similarly, our results showed that serum sodium was significantly lower in children with perforated AA.

We also detected and compared the levels of DLAC (specific bacterial metabolism), PCT (a biomarker of bacterial infection), bilirubin, AST, and ALT between perforated and nonperforated groups. The results suggested that the above markers had no significant difference between groups.

## 5. Conclusions

In summary, our data suggested that children with perforated AA often presented with significantly longer duration of symptoms, higher white blood cell count, CRP level, neutrophils percentage, and lower serum sodium level. Logistic regression analysis showed that elevated white blood cell count (>12000 m^3^) with CRP level (>8 mg/dL) and elevated neutrophils percentage (>74%) with CRP level (>8 mg/dL) were associated with risk of perforation. A clinician should have a higher index of suspicion for perforated AA in a pediatric patient presenting with elevated white blood cell count with CRP level and elevated neutrophils percentage with CRP level.

## Figures and Tables

**Table 1:** Demographic characteristics of children with acute appendicitis.

	Total (*n* = 1895)	Perforated (*n* = 613)	Nonperforated (*n* = 1282)	*P* value
Age (years)	8 (3–18)	5 (3–18)	9 (3–18)	0.001
Female-male ratio	1 : 1.11	1 : 1.19	1 : 1.08	0.23
Temperature	38.1 ± 0.7	37.9 ± 0.8	38 ± 0.9	0.46
Body mass index (kg/m^2^)	19.67 ± 0.38	19.21 ± 0.46	19.98 ± 0.55	0.53

Values are presented as mean ± standard deviation. The temperature is measured in Celsius.

**Table 2:** Symptoms and signs of children with acute appendicitis.

	Total (*n* = 1895)	Perforated (*n* = 613)	Nonperforated (*n* = 1282)	*P* value
Migrating pain	371 (19.6%)	105 (17.2%)	277 (21.6%)	0.28
Fever	265 (14%)	99 (16.1%)	169 (13.2%)	0.33
Nausea	487 (25.7%)	175 (28.6%)	287 (22.4%)	0.71
Vomiting	591 (31.2%)	169 (27.5%)	433 (33.8%)	0.68
Diarrhea	502 (26.5%)	191 (31.2%)	319 (24.9%)	0.7
Anorexia	116 (6.1%)	35 (5.7%)	82 (6.4%)	0.43
Right lower quadrant tenderness	1724 (91%)	566 (92.3%)	1150 (89.7%)	0.23
Right lower quadrant rebound tenderness	1380 (72.8%)	412 (67.2%)	1001 (78.1%)	0.11
Duration of symptoms >24 h	817 (43.1%)	418 (68.2%)	379 (29.6%)	**0.001**
Time from admission to surgery (h)	10.7 (5.9–12)	11 (5.7–14)	10.2 (6.3–13)	0.23

Categorical values were expressed as *n* (%), and continuous values were expressed as median (interquartile range). Bold denotes significant *P* value less 0.05.

**Table 3:** Laboratory markers in children with acute appendicitis.

	Perforated (*n* = 613)	Nonperforated (*n* = 1282)	*P* value
WBC (mm^3^)	14,890 ± 723	12,650 ± 558	**0.03**
CRP (mg/dL)	8.37 ± 0.84	5.79 ± 0.65	**0.01**
Neutrophils (%)	77.3 ± 1.5	65.1 ± 1.4	**0.02**
Platelet count (×10^9^/L)	312 ± 119	327 ± 86	0.34
Serum sodium (mEq/L)	133 ± 2	137 ± 2	**0.001**
PCT (ng/mL)	0.8 ± 1.2	0.7 ± 0.9	0.43
DLAC (mmol/L)	0.3 ± 0.2	0.4 ± 0.5	0.23
Bilirubin (mg/dL)	0.85 ± 0.03	0.87 ± 0.02	0.27
AST (U/L)	22.8 ± 0.6	22.3 ± 0.5	0.53
ALT (U/L)	14.7 ± 0.6	15.1 ± 0.4	0.23

Values are presented as mean ± standard deviation. WBC, white blood cell; CRP, C-reactive protein; PCT, procalcitonin; DLAC, D-lactate; AST, aspartate aminotransferase; ALT, alanine aminotransferase. Bold denotes significant *P* value less 0.05.

**Table 4:** Risk factors of perforated appendicitis in children.

Factors	*P* value	Adjusted OR (95% CI)
Duration of symptoms >24 h	0.34	0.98 (0.83–1.25)
WBC (>12000 mm^3^)	0.06	1.09 (0.67–1.85)
CRP (>8 mg/dL)	0.12	1.36 (0.71–1.92)
WBC (>12000 mm^3^) + CRP (>8 mg/dL)	**0.001**	**4.62 (2.56–6.23)**
Neutrophils (>74%)	0.13	1.21 (0.53–1.77)
Neutrophils (>74%) + CRP (>8 mg/dL)	**0.001**	**5.67 (3.79–8.54)**
Serum sodium (<135 mEq/L)	0.21	0.78 (1.45–3.61)

WBC, white blood cell; CRP, C-reactive protein. Bold denotes significant *P* value less 0.05.

## Data Availability

The data used to support the findings of this study are available from the corresponding author upon request.
